# Depression in patients with severe somatic disease – study protocol of the prospective DESIE-study

**DOI:** 10.1186/s40359-025-03810-w

**Published:** 2025-12-09

**Authors:** Simone Fischer, Dennis Freuer, Lino Braadt, Michael Ertl, Elisabeth Schnoy, Johanna Maria Classen, Felix Müller, Matthias Wahle, Violetta Rakina, Yvonne Gosslau, Astrid Röh, Alkomiet Hasan, Jakob Linseisen, Christine Meisinger

**Affiliations:** 1 Epidemiology, Medical Faculty, University of Augsburg, Stenglinstr. 2, Augsburg, 86156 Germany; 2 Department of Neurology and Clinical Neurophysiology, University Hospital Augsburg, Augsburg, Germany; 3 Department of Gastroenterology and Infectious Diseases, University Hospital Augsburg, Augsburg, Germany; 4 Division of Rheumatology & Clinical Immunology, Department of Medicine 3, University Hospital Augsburg, Augsburg, Germany; 5 Department of Vascular Surgery, University Hospital Augsburg, Augsburg, Germany; 6 Department of Psychiatry, Psychotherapy and Psychosomatics, Faculty of Medicine, University of Augsburg, Augsburg, Germany; 7 DZPG (German Center for Mental Health), Partner Site München/Augsburg, Augsburg, Germany; 8 Institute for Medical Information Processing, Biometry and Epidemiology - IBE, LMU Munich, Munich, Germany

**Keywords:** Comorbid depression, Cardiovascular disease, Systemic inflammatory disease, Cohort study, Mental health

## Abstract

**Background:**

Depression is a big health concern and a leading cause of disability worldwide that is inadequately addressed. Severe somatic illnesses are often associated with psychological distress that can reach the level of a depressive disorder requiring treatment. Depression as a comorbidity is linked with a poor quality of life and noncompliance to treatment in these patients, which may lead to increased use of health services. Information on the frequency and the disease course of depression in patients with severe somatic diseases and the need for inpatient treatment and subsequent treatment is still lacking.

**Methods:**

A cohort study including patients with a diagnosis of cardiovascular diseases (heart attack, stroke, peripheral artery disease) or systemic inflammatory disease (rheumatoid arthritis, inflammatory bowel disease) who are admitted to the University Hospital of Augsburg will be established. At recruitment during hospital stay/outpatient visit, data on mental health (e.g. depressive symptoms, stress, quality of life), clinical data, and lifestyle data will be gathered through self-administered questionnaires and chart review. Furthermore, blood samples will be collected and stored. After hospital discharge, study participants will be repeatedly contacted over time (at the beginning in shorter intervals, then about every 3 months) to provide further information about their mental well-being, life circumstances (e.g. incapacity to work), quality of life, and utilization of medical services. Altogether at least 500 patients should be included and followed up in this study. Descriptive statistics including prevalence and group differences will be calculated. Multivariable generalized linear mixed-effects models with random intercepts will be performed to examine determinants of depression and predict the occurrence and course of the disease.

**Discussion:**

The strengths of the DESIE study are the mainly digital assessments and its longitudinal character with frequent follow-up questionnaires. The study will substantially contribute to the current research about comorbid depression in somatic diseases.

**Trial registration:**

The study was prospectively registered in the German Clinical Trials Registry and WHO International Clinical Trials Registry Platform (registration number: DRKS00033245, date of registration: 14 December 2023) prior to the start of recruitment.

## Background

Depressive disorders are a major public health problem both nationally and internationally, because of its relatively high prevalence and its association with significant disability [1, 2]

It is well known that numerous somatic illnesses are associated with depression. In a large World Health Survey including more than 240,000 participants from 60 countries, an average between 9.3 and 23.0% of participants suffering from at least one chronic somatic disease had comorbid depressive symptoms [2].

Approximately half of the adult population lives with one or more chronic diseases [3]. Many of them must cope with a range of challenges, such as difficult symptoms or loss of independence; even daily self-management could become a hurdle. Due to those challenges, people with chronic disease are at significantly increased risk of depression, compared to the general population [4, 5]. In addition, the co-occurrence of psychological distress in chronic disease is associated with a range of adverse outcomes, such as poorer self-rated health [6] increased work absenteeism [7], and poorer disease self-management and prognosis, including lower adherence to treatments and lifestyle recommendations [8].

Thus, in the context of somatic diseases, depression is of particular importance. Especially severe somatic diseases that are accompanied by inflammatory processes seem to increase the risk of depression [9]. Besides biological mechanisms (e.g., inflammation) [10], also psychological mechanisms (e.g., fear of the consequences of the somatic illness) or associated circumstances (e.g., the need to attend repeated appointments and examinations due to the somatic disease) may significantly promote the manifestation of depression.

In the context of the widespread diseases stroke and myocardial infarction, depression usually occurs after the acute event. Currently, there are only few options to support these patient groups. For example, the point prevalence of major depression after myocardial infarction is reported to be 28.7% [11]. Prevalence rates of depression are influenced by the severity of the underlying somatic disease [12]. A large cohort study of 157,243 stroke patients found that 25.4% of patients developed depression within two years of study entry compared with only 7.8% in the reference population [12]. However, also, patients with other physical disorders, such as peripheral artery disease [13], cancer [14] and autoimmune diseases [15, 16] show substantially higher frequencies of depressive disorder than observed in the general population.

Often, depressive disorders are diagnosed too late and thus also worsen the prognosis of the somatic disease. Depression impairs quality of life and social participation, leads to immense direct and indirect costs (especially sick days and reduced earning capacity), and places a burden on sufferers and their families. In addition, a large-scale meta-analysis based on 48 studies and 55,898 people showed that comorbid depression in somatic illness significantly increases treatment costs (16 studies, 1.39 95%CI 1.24—1.55, *p* < 0.001) [17].

There is evidence that psychotherapy and psychosocial approaches successfully reduce distress associated with chronic illness [18], thereby promoting successful adjustment. However, these treatments appear to be effective only in people who meet the criteria for a depressive disorder, as opposed to unselected samples [18]. Importantly, many patients appear to have increased depressive or anxiety symptoms after the onset of a chronic illness that resolves on its own (e.g., [19, 20]). This fits with the concept of psychological adjustment as a return to equilibrium after a stressor, with people using their resources to achieve a "new normal" [21, 22]. In such cases, psychological treatment may not be clinically necessary and thus not cost-effective. However, it is not clear what proportion of people with chronic illnesses consistently experience increased distress and when in the course of the disease the need for psychological treatment arises. A clear understanding of the proportion and timing of patients' psychological adjustment is important for the appropriate implementation and the success of psychological intervention in our health care systems.

Epidemiological studies suggest that fewer than 20% of individuals with depression are adequately treated [23]. The resource of professionals for timely diagnosis and treatment is limited [24, 25] leading to a situation of undersupply. In addition, due to demographic change, an increase in somatic diseases and, as a consequence, an increase in depression is to be expected [26]. Therefore, a major challenge in the coming years is to improve the care of people with mental illnesses, especially depression.

Data from Germany regarding the diagnosis and care of depression in people with a severe somatic disease is still scarce [27, 28]. In addition, none of these prior studies prospectively investigated the risk of depression in patients with somatic diseases.

The prospective DESIE study will investigate the frequency and the disease course of depression in patients with severe somatic diseases, that is myocardial infarction, stroke, peripheral artery disease or systemic inflammatory diseases. The main aim of the project is to identify patients who are already showing signs of depression at an early stage. A special feature of the DESIE study is that, unlike existing cohorts, it will collect data digitally at baseline and during follow-up. Based on the available data (personal characteristics, lifestyle data, clinical data, biomarker data), determinants will be analyzed; an algorithm will be developed to predict the development of depression and its course in each somatic disease entity.

As secondary research questions the acceptance of digital medicine in this patient group, health care utilization of patients with severe somatic diseases with and without mental health problems, and additional psychological impairments will be investigated.

## Methods

The present study is part of the digiBRAVE (digitales Bayerisches (Früh-)Diagnostik-, Präventions- und Therapieprogramm Depression) project at the University Hospital of Augsburg and the Bezirkskrankenhaus Augsburg with the aim to promote health and reduce the burden of disease through targeted digital prevention, diagnosis, and treatment of concomitant depression in somatic disease. The project will involve collaboration between different disciplines, namely digital medicine, psychiatry and psychotherapy, neurology, ethics, general medicine and epidemiology. It is funded by the Bavarian State Ministry of Health and Care.

The DESIE study will include patients who are hospitalized or visiting a clinical outpatient department due to a serious somatic disease at the University Hospital Augsburg. The focus of the present study is on the inclusion of patients with stroke, myocardial infarction, peripheral artery disease, and systemic inflammatory diseases (chronic inflammatory bowel diseases and rheumatoid arthritis). However, patients with other severe diseases (e.g. cancer, long-covid) may be included if they wish. Participation in the study is voluntary, irrespective of management strategy and outcome. If a patient is willing to take part in the study, a written informed consent form has to be signed by him/her. The data collection will be performed in accordance with the Declaration of Helsinki. The data underlies professional discretion and data protection according to the General Data Protection Regulation (GDPR). Personal identifying data and scientific data are stored separately under pseudonyms. Information on data protection is included in the written patient information and the consent forms.

At the University Hospital Augsburg trained study nurses prospectively record all cases of heart attacks, strokes, peripheral artery diseases, and systemic inflammatory diseases and visit the patient to inform her/him about the study and to deliver the study documents. Patients will receive comprehensive and understandable information on the processes and consequences of participation in the study. It is planned to recruit at least 500 patients during the project.

The required sample size was calculated, expecting a prevalence of 20% of depressive symptoms in patients with somatic diseases and a given type I error of 0.05. Depending on a specific determinant of interest (e.g. childhood trauma, level of education, perceived stress) with a true probability of at least 15% for depressive symptoms, the sample size needs to be at least 380 to achieve a statistical power of at least 0.8. Considering the comprehensive questionnaires and frequent follow-up time points, a drop-out rate of 25% can be expected based on previous experience with longitudinal studies. Therefore, the recruitment of at least 500 patients should be sufficient to answer several research questions. The sample size calculations were performed with the software PS (Power and Sample Size Calculations, Version 3.0).

Based on the experience in previous comparable studies, it is expected that this project aim can be reached. The recruitment phase is planned stepwise (staggered start of recruitment for the different disease entities) and will continue until May 2025. Recruitment of patients began in January 2024.

Inclusion criteria.

- Inpatient admission or visit at the clinical outpatient department of the University Hospital Augsburg
- Age 18 years and older
- Capable of giving informed consent
- Sufficient fluency in German to understand the implications of participating in the study
- Admission diagnosis (suspected diagnosis) of:
  - Vascular disease such as stroke, peripheral artery disease or myocardial infarction (core group)
  - Systemic inflammatory disease (core group)
  - Other serious somatic diseases (e.g., cancer, long-covid), if they wish to participate in the study

Exclusion criteria.

- Lack of capacity to give consent (e.g. dementia, language difficulties)
- Age < 18 years
- No interest in participating in the study

Patients with a physician diagnosis of depression prior to the hospital stay/outpatient visit will not be excluded from the study.

### Data collection at baseline hospital stay or visit at clinical outpatient department

After patients have signed the informed consent form, study nurses will hand over a standardized questionnaire during their hospital stay or a visit at a clinical outpatient department (either in the form of an online questionnaire via tablet computer or in paper–pencil form). The questionnaire will cover via a self-rating demographic information, comorbidities, lifestyle data (including physical activity), disease history, the PHQ-9 (Questionnaire to assess depressiveness) [29], the WHO-5 (World Health Organization (WHO) index of well-being) [30],

WHOQOL-BREF (quality of life) [31], the EQ-5D (EuroQol-5D) questionnaire [32], the Childhood Trauma Questionnaire (CTQ) [33], the Perceived Stress Questionnaire (PSQ) [34], questions about the acceptance of digital medicine (ADM) [35], subjective experience of discrimination (SDE), and self-stigmatization (SSMI questionnaire) [36]. Routinely collected clinical data on comorbidities, risk factors, medications prescribed, diagnostic procedures, clinical characteristics, laboratory parameters, and invasive and non-invasive treatment regimens as well as complications during hospital stay will be assessed by chart review. The decision of focusing on self-administered questionnaires has been made to facilitate recruitment and to allow for remote assessments during the follow-up periods.

### Follow-up surveys

All study participants are followed up at specific time points after being discharged from the hospital (see Table 1). Follow-up surveys can also be conducted online (survey link is sent via e-mail) or in paper–pencil form. Some of the questionnaires from the baseline survey are also used in the follow-up surveys. In addition, questions are asked about days of incapacity to work (if still working), visits to the doctor, inpatient admissions and return to work/training/education. After initially close follow-up time periods, the plan calls for surveys to be conducted every three months up to one year. Participants receive a reminder to complete the questionnaire via e-mail or phone call after three to seven days depending on the number of the follow-up survey.

**Table 1:** Data collection as part of the DESIE study

Time points of data collection	T_0_ (Baseline)	T_1_	T_2_	T_3_	T_4—6_
Days	During hospital stay	14	28	90	every 90
PHQ-9 (patient health questionnaire)	X	X	X	X	X
WHO-5 (WHO index of well-being)	X	X	X	X	X
EQ-5D questionnaire	X	X	X	X	X
WHOQOL-BREF (quality of life)	X	X	X	X	X
PSQ (Perceived Stress Questionnaire)	X	X	X	X	X
CTQ (Childhood Trauma Questionnaire)	X				
Sociodemographic data	X				
Disease history	X				
Data on lifestyle	X				
ADM (acceptance of digital medicine)	X			X	
SDE (subjective experience of discrimination)	X				
SSMI (Self-stigmatization)	X				
Days of incapacity to work*			X	X	X
Doctor visits			X	X	X
Hospitalizations			X	X	X
Return to work/training place*			X	X	X

^*^if still employed or in training

### Collection of blood

During the hospital stay about 30 mL blood will be collected from the patients of the core group, that is patients with stroke, heart attack, peripheral artery disease, or systemic inflammatory diseases. If possible, blood samples will be taken as part of the routine blood tests scheduled for patients during their inpatient stay or outpatient appointments. Laboratory staff at the Chair of Epidemiology will be in charge of processing and aliquoting of the samples, and the storage of the sample tubes at −80 °C in the freezers at the Chair of Epidemiology.

### Patients with depressive symptoms

Generally, study participants are offered a leaflet with information about depression and where to get help. If patients show noticeable depressive symptoms during their hospital stay and study participation, the study nurses inform the attending physician for a psychiatric consultation including suicidality risk assessment. In addition, a digital stepped care program with digital services that can be individually tailored to the severity and intensity of depressive symptoms is concurrently developed for these patient group as a part of the superordinate project digiBRAVE.

If baseline data from participants with stroke indicate relevant symptoms of depression (PHQ-9 ≥ 10 points) a more detailed evaluation will be performed by study personnel. These individuals are immediately offered the opportunity to participate in an intervention study with tele-psychological treatment (DISCOVER study) [37], which is not part of this cohort study. All patients who accept the invitation to the intervention study must sign separate informed consent forms.

### Statistical analysis

After data cleaning, quality assurance, and transformation of variables descriptive statistics and explorative data analysis will be carried out.

The prevalence of psychological impairments and baseline characteristics will be reported by calculating appropriate summary statistics. Depending on their distributions, continuous variables will be presented either as means and standard deviations or medians and interquartile ranges. Where the normality assumption is met, differences between independent groups will be preferably tested using two-sided parametric statistical tests (i.e. *t*-test, ANOVA). Otherwise, equivalent non-parametric tests will be used (i.e. Mann–Whitney-U test, Kruskal–Wallis test). Considering re-measurements in the longitudinal design, corresponding parametric and non-parametric tests will be performed (i.e. paired *t*-test, Wilcoxon signed-rank test, repeated measures ANOVA, and Friedman’s test). If there are notable differences in more than two groups or time points, additional appropriate post-hoc tests will be conducted. Pairwise comparisons of categorical variables will be assessed using Fisher's exact test, while differences in frequencies over time will be tested with the Cochran’s Q test. Assumptions of normality, sphericity, and equal variances will be ensured performing Shapiro–Wilk test, Mauchly’s test, and Levene’s test, respectively.

The regression analyses have two goals, first to examine determinants of the onset of depression following major somatic diseases, and second to predict the occurrence and course of the disease. Concerning the dependency structure in the longitudinal design, multivariable generalized linear mixed-effects models with random intercepts will be performed in both association and predictive analyses. All necessary model assumptions will be ensured. In particular, non-linear relationships between continuous independent variables and the potentially transformed outcome will be modelled using restricted cubic splines. The variance inflation factor (VIF) will be calculated to assess potential multicollinearity. Effect modification by age and sex will be examined by adding interaction terms to the regression models. In the association analyses, confounders will be selected based on directed acyclic graphs (DAGs) that consider medical and biological causal structures and account for instruments, mediators, and colliders. The assumed associations to build the DAGs will be based on a literature review of the current state of research. If necessary, different sets of confounding will be used in sensitivity analyses including additional assessment of unmeasured confounding (e.g. E values). The effect of potentially missing data in follow-up questionnaires will be taken into account through sensitivity analyses and, where appropriate, suitable imputation methods.

Data-driven development of the diagnostic algorithm will be carried out on a training data set. Main metrices of model performance are parameters of discrimination such as the Area under the curve (AUC) within the framework of a ROC analysis and "concordance” statistic (C statistics). Ten-fold cross validation on different subsets of the data sets will be used for internal validation. Moreover, calibration plots are used to ensure a well-calibrated prediction algorithm. Finally, the algorithm will be tested on a training data set (subset validation).

All statistical tests within the multivariable models will be conducted at an alpha level of 0.05. A correction of the significance level alpha will be made in case of multiple testing. Statistical analyses will be performed using SAS or R software applications.

### Dissemination

The study was registered in the German Clinical Trials Registry (study ID: DRKS00033245) in December 2023 prior to the start of recruitment and will be updated accordingly. Findings from the DESIE study will be presented at academic conferences and published in peer-reviewed scientific journals.

## Discussion

The DESIE study is a prospective, observational cohort study in which patients with severe somatic diseases (heart attack, stroke, peripheral artery disease, systemic inflammatory disease or other diseases) are recruited and their characteristics and outcomes measured longitudinally. The focus will lay on the digital assessment of mental health outcomes with the aim of implementing a mainly digitalized follow-up. Based on the data obtained during the project, an algorithm will be developed to predict the development of depression as it progresses. In addition, the care of patients with depression in chronic somatic diseases is to be improved.

The strength of this study is a comprehensive mainly digital baseline assessment, the collection of blood samples and its longitudinal character with frequent, mainly digital, follow-up questionnaires. However, the follow-up assessments can also be a limitation if a lot of participants drop out of the study. To overcome this limitation patients are informed comprehensively about the frequency and timing of the follow-ups and are reminded via e-mail or phone contact to complete the questionnaire. Another limitation is that patients with e.g. severe stroke may be not able or refuse to complete the questionnaire during their hospital stay due to physical or mental impairments which can lead to a selection bias towards less severe cases. Due to the very intimate and subjective nature of the topics, we did not consider proxy interviews (e.g. by a relative) to be appropriate, but the study nurses offer assistance to impaired patients and provide a shorter version of the baseline questionnaire if necessary. Furthermore, the study is planned as a single-centre study which limits generalizability as participants from one university hospital may differ from patients of other regions. However, after the initial funding phase, the study design may be expanded to further university hospitals in the future.

Overall, the DESIE study will make substantial contributions to the current knowledge about comorbid depression in somatic diseases. Based on the study results of DESIE, in combination with the superordinate project digiBRAVE involving various disciplines, screening, prevention, and treatment of comorbid depression may be enhanced.

## Data Availability

Not applicable.

## Abbreviations

ADM: acceptance of digital medicine
CTQ: Childhood Trauma Questionnaire
DAG: Directed acyclic graph
DESIE: Depression bei schweren somatischen Erkrankungen (english: Depression in severe somatic diseases)
digiBRAVE: Digitales Bayerisches (Früh-)Diagnostik-, Präventions- und Therapieprogramm Depression (english: digital Bavarian program for screening, prevention and therapy of depression)
DISCOVER: Depression-intervention study for optimization of reconvalesence after stroke
EQ-5D: EuroQol five dimensions
PHQ: Patient Health Questionnaire
PSQ: Perceived Stress Questionnaire
QoL: Quality of life
ROC: Receiver operating characteristic
SDE: Subjective experience of discrimination
SSMI: Self-Stigma of Mental Illness
WHO: World Health Organization
